# Spatial Associations Between Land Use and Infectious Disease: Zika Virus in Colombia

**DOI:** 10.3390/ijerph17041127

**Published:** 2020-02-11

**Authors:** Joshua S. Weinstein, Timothy F. Leslie, Michael E. von Fricken

**Affiliations:** 1 Geography and Geoinformation Science Department, George Mason University, 4400 University Drive, Fairfax, VA 22030, USA; tleslie@gmu.edu; 2 Department of Global and Community Health, George Mason University, 4400 University Drive, Fairfax, VA 22030, USA; mvonfric@gmu.edu

**Keywords:** zika virus, ZIKV, Colombia, infectious disease, vector-borne, land use, landscape metrics, linear density, proportion abundance, patch density

## Abstract

Land use boundaries represent human–physical interfaces where risk of vector-borne disease transmission is elevated. Land development practices, coupled with rural and urban land fragmentation, increases the likelihood that immunologically naïve humans will encounter infectious vectors at land use interfaces. This research consolidated land use classes from the GLC-SHARE dataset; calculated landscape metrics in linear (edge) density, proportion abundance, and patch density; and derived the incidence rate ratios of the Zika virus occurrence in Colombia, South America during 2016. Negative binomial regression was used to evaluate vector-borne disease occurrence counts in relation to Population Density, Average Elevation, Per Capita Gross Domestic Product, and each of three landscape metrics. Each kilometer of border length per square kilometer of area increase in the linear density of the Cropland and Grassland classes is associated with an increase in Zika virus risk. These spatial associations inform a risk reduction approach to rural and urban morphology and land development that emphasizes simple and compact land use geometry that decreases habitat availability for mosquito vectors of Zika virus.

## 1. Introduction

Worldwide, Colombia has the highest proportional burden of Zika virus (ZIKV) cases [1]. While only 8.7 percent the total area of the contiguous United States, Colombia experienced greater than 2000 times the lab confirmed ZIKV cases as the United States in 2016 [2]. In addition to the adverse impact on health, public concern of infection resulted in a 70 percent economic decline in tourism to Colombia during the first half of 2016 [3].

Transmission of ZIKV occurs primarily through the bite of an infected Aedes mosquito, specifically Aedes aegypti and Aedes albopictus, but can also be transmitted sexually. Aedes spp. mosquitoes have adapted to live and breed in a variety of habitats. Historically, the preferred breeding habitats of Aedes spp. are ground or natural pools, both salt and fresh water [4]. However, these mosquitoes have adapted to using containers, tires, and plant pots as breeding grounds and are now found in urban, rural, and forested areas [4,5,6]. This adaptation is believed to be the reason behind the international spread of Aedes spp. mosquitoes to over six continents [4].

A study by Ali et al. [7] suggests the drivers of ZIKV in the Americas are land use change, climate variation, poverty, and human movement. Combined with Aedes spp. ability to adapt to new breeding habitats, changes in anthropogenic land use through urbanization, agricultural practices, and deforestation assisted with the emergence of ZIKV across the Americas. Human contact with infected vectors near local land use boundaries is a key driver of vector-borne disease (VBD) emergence [8,9,10,11]. Land use change and fragmentation increases the frequency of human-mosquito contact and alters the dynamics of pathogen–vector–host relationships by providing more opportunities for pathogens to expand their geographic range and exploit new habitat niches [12,13]. In short, changes in anthropogenic land use due to urbanization, changing agricultural practices, and deforestation has led to the reemergence of arboviruses, such as ZIKV, across the Americas. This is complicated further due to the limited information available regarding potential wildlife reservoirs exposed to ZIKV through reverse zoonosis and what role they play in disease maintenance along land use boundaries.

The proliferation of container breeding sites has facilitated the adaptation of A. aegypti and A. albopictus, which, in turn, has resulted in an increased incidence of ZIKV, dengue, and other arboviruses in Latin America [5]. The influence of land use on VBDs has been well documented. Vanwambeke et al. [14] discovered an association between land use and dengue risk for A. albopictus in Hawaii. Another study by Lambin et al. [15] found a strong association between West Nile virus and the density of land use boundaries produced by fragmentation in southern France.

In Cote d’Ivoire, the prominence of artificial containers was the primary contributor to the increase of Aedes larvae in urban settings [6]. These changes result in new environments that are optimal for mosquito breeding, including areas which were previously uninhabitable [16]. Furthermore, these new habitats tend to be in close proximity or embedded within human settlements, increasing the risk of infection due to more frequent interactions [11,17,18,19].

Land use resulting in deforestation or agricultural development leaves human inhabited areas in close contact with forested or cropland areas. A study done in Brazil looked at the relationship between Aedes spp. mosquitoes, specifically Aedes albopictus, and the interface between urban areas and the forest edge. The study found that vector density was highest at the forest edge and decreased significantly only 200–300 m into the forest. As the forest edge was close to an urban area, the study identified 75% of engorged mosquitoes at the forest edge as having fed from a human host rather than animal hosts [20]. A similar study done in Rio de Janiero, found an absence of Aedes aegypti or Aedes albopictus 100 m into the forest, again showing that Aedes spp. mosquitoes prefer the edge of forested areas [21].

Complex dynamics between pathogen reservoirs, vectors, and host behaviors influence emergence likelihood, making the task of targeted VBD prevention and mitigation challenging [22]. While the spatial associations between land use and VBD is not novel, the majority of research to date has occurred via localized field research using mosquito trapping rather than county- or region-wide research linked with lab confirmed case counts and remote sensing data. Understanding the spatial associations between the density of land use boundaries and ZIKV occurrence can help policy makers and public health stakeholders identify potential hotspots of transmission and thus mitigate local outbreaks before they grow in scale [12,23]. The objective of this research was to expand the spatial scope beyond localized field studies to the country-level using lab confirmed case counts and remotely sensed derived data to identify associations between ZIKV occurrence and the density of land use boundaries in Colombia during the 2016 outbreak.

## 2. Methods 

We obtained ZIKV lab confirmed case count, land use, and population data from 2016. Scale challenges inherent to openly available data restricted research to the 2nd order administrative area, the Colombian municipality (equivalent to the United States “county”). Additionally, the infrequency of land use dataset creation restricted research to the use of 2014 produced land use data.

### 2.1. Case Data

The GitHub data repository provides publicly available ZIKV incidence data across a breadth of Central and South American countries through the translation of and content extraction from Epidemiological Bulletins created by local-level health departments or ministries. Municipality-level weekly Epidemiological Bulletins published by the Colombian Instituto Nacional de Salud (National Institute of Health) was the original source used to create the data available through GitHub [24]. Although contributors to this repository claim the data is not exhaustive or official, it was the most spatially granular and comprehensive publicly available and centralized data repository of ZIKV cases. Arsenault et al. [25] identified municipality as the appropriate scale for VBD research within Quebec, Canada. Their research tested nine VBD related factors, to include data access and the distribution of cases and population, across a variety of area types, such as naturally occurring watersheds and artificially designated boundaries. The researchers determined that their nine factors were optimized at the municipality level. Similarly, the municipality within Colombia—the second-order administrative unit—was an appropriate scale for ZIKV research. Data at a more granular scale was not publicly available. Further, additional granularity would have increased the likelihood that health report locations fell outside of the unit where disease transmission took place. Conversely, aggregation of data into units larger than second-order would have introduced the modifiable areal unit problem, whereby spatial detail and insight is lost.

### 2.2. Land Use Data Collection

Land use data for this study in Colombia was derived from Global Land Cover-SHARE (GLC-SHARE), a best-of-breed, centralized, mosaicked, and harmonized database of regionally produced land use datasets. GlobCover 2009, Moderate Resolution Imaging Spectrometer (MODIS) 2010, and Cropland Database 2012 supplement GLC-SHARE in areas where higher resolution authoritative national or regional data is not available, resulting in a dataset with a resolution of at least 1-km per pixel. Fitness-for-use for GLC-SHARE sources are determined through imagery and ground-truth comparison with the best available source determined at the pixel level [26]. Consolidation of land use classes considered duplicative within the lens of this research topic reduced analytic and computational load [27]. As such, the GLC-SHARE classification schema and data dictionary guided the mapping and consolidation of the 11 GLC-SHARE land use classes into 8 Research Classes (RCs), as depicted in Table 1.

The RCs Cropland, Grassland, and Tree-Covered account for more than 98 percent of all land use within Colombia—74 percent for Tree-Covered, 19 percent for Grassland, and 5 percent for Cropland. Land use data came from the Food and Agriculture Organization of the United Nations GLC-SHARE dataset. Environmental and physiographic variables such as temperature [28], rainfall [29], and elevation [30] are associated with VBD. Collinearity was substantial between these three variables, and as average temperature and rainfall data was not available for Colombia at or more granular than the second-order administrative unit since the 1980s, this research made use of average elevation from the National Aeronautics and Space Administration’s Shuttle Radar Topography Mission. Average Per Capita Gross Domestic Product (GDP) from DANE, the Colombian National Statistics Office, was normalized for the spatial association for an economic variable [31]. Lab-confirmed ZIKV occurrences were pulled from GitHub [24]; population counts from the Colombian National Department of Statistics [32]; and municipality boundaries from the Database of Global Administrative Areas [33]. All data used in this research were free, open source, and exempt from human subjects’ review.

### 2.3. Land Use Calculations

The calculation of land use boundary density provides a landscape metric akin to fragmentation, and one in which the spatial unit of the linear density calculation is equal to that of the ZIKV dataset, the second-order administrative unit. To calculate land use boundary density, Geographic Information System functionality was used to intersect RC and administrative areas. Following this, geometry calculations derived RC boundary length within each administrative unit. Dividing boundary length by area determined the RC linear density (length per area). The following equation calculates the linear, or edge, density:

(1) $$Linear\;Density=\frac{\sum_{i=1}^{n}L_{i}}{A},$$

where *L* = length of boundary within administrative unit, *A* = area of administrative unit.

**Figure 1:**
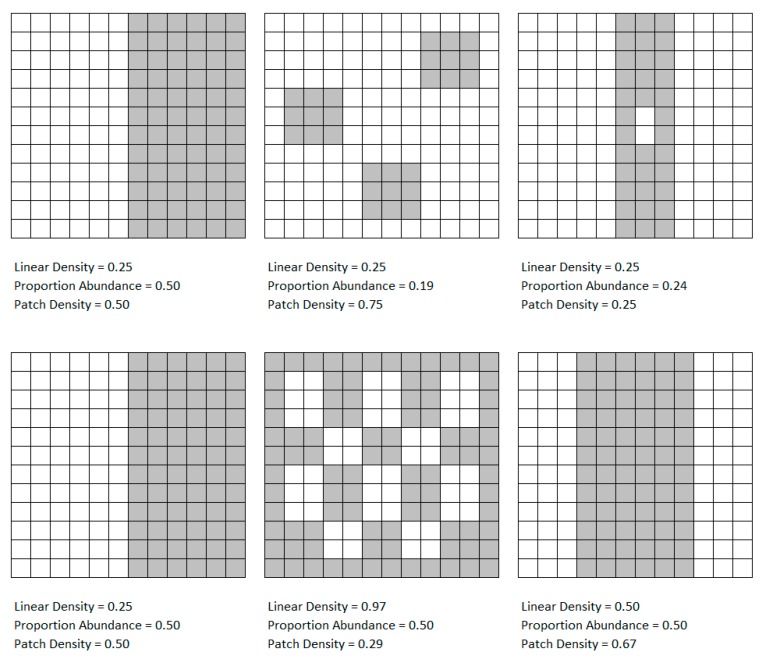

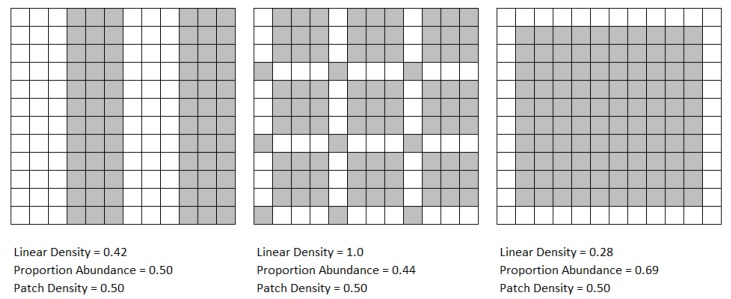
Relationship between linear density, proportion abundance, and patch density.

Proportion abundance is a landscape metric that measures the area of a single RC compared to the area of all RCs in the same second-order administrative unit. Patch density is a landscape metric that measures patch count of a single RC in a unit compared to the total patch count of all RCs in the same unit. An increase or decrease in proportion abundance or patch density landscape metric does not necessarily result in a corresponding increase or decrease in the linear density of land use boundaries. The proportion abundance and patch density landscape metrics can, however, provide context to the linear density metric and its association with VBD occurrence. Figure 1 depicts the relationship between the three landscape metrics. The linear density of the gray patches within the first row remain constant, as does proportion abundance in the second row, and patch density in the third row.

### 2.4. Statistical Analysis

**Figure 2:**
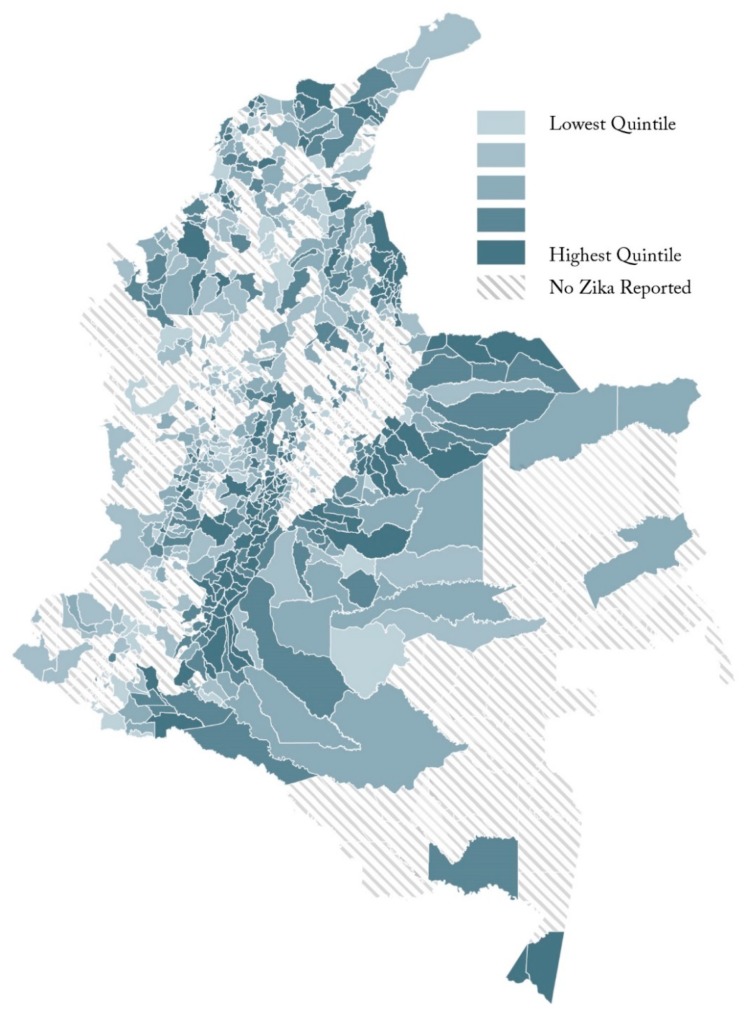
Municipality-level Zika virus (ZIKV) occurrence per capita.

VBD occurrence counts served as the dependent variable of our analysis. In Colombia during 2016, forty-five percent (509 out of 1121) of the municipalities did not experience a single ZIKV occurrence, as depicted in Figure 2. The lab-confirmed occurrence count of 501,970 in a population of 48,881,635 results in an occurrence rate of 1 percent, or 1027 out of 100,000. With the count data, as well as the overdispersion of the variable compared to its mean, we employed negative binomial regression to model the association between ZIKV count and a number of potential drivers of disease occurrence [34]. 

Several independent variables were included in our analysis. Our main variables of interest are the three landscape metrics described above. While we initially planned to use the top three RCs, because of substantive cross-correlation to the three-covered metrics, we simplified the analysis to only include Cropland and Grassland. The choice to remove Tree-Covered land use, despite being the most prevalent land cover, was due to behaviors in the region focusing on increasing grassland and cropland through deforestation [35]. 

We first modeled the effects of each land use metric separately, and then also one with all metrics combined (four models total). For each model, we control for the regional population density [36], per capita GDP [37], and elevation [38], scaling population density by 100 and the latter two variables by 1000 to assess the significance of substantive change. The correlation matrix of the model variables is included as Supplementary Table S1.

Table 2 presents a descriptive summary of the variables included in building the statistical models. Further, variable information spans each landscape metric model—Linear Density, Proportion Abundance, and Patch Density—for each RC in Cropland and Grassland.

## 3. Results

We present the incidence rate ratios (IRR) produced by these models in Table 3. Each landscape metric is tested by itself along with one model that incorporates all three landscape metrics. All four models had significant likelihood ratio chi-square values. Akaike information criterion (AIC) values indicated a slight benefit for the full model (Model 4) over each of the models of just a single land use metric. 

The land use metrics for the RCs were significant in almost all situations across the models. Increasing the linear density by a kilometer of border length per square kilometer of cropland and grassland was associated with a 0.1 and 0.6 percent increase in ZIKV, respectively. Increases to the proportion abundance of cropland decreased the cases of ZIKV, while increases in the abundance of grassland were inconsistent in effect across the two models (one a small, but significant increase, the other insignificant). Patch density showed a similar trend, with increases in the patch density of cropland having an inconsistently negative effect. For every percent increase of the patch density of grassland, there was a 2.3 to 3.5 percent increase in reported ZIKV.

**Figure 3:**
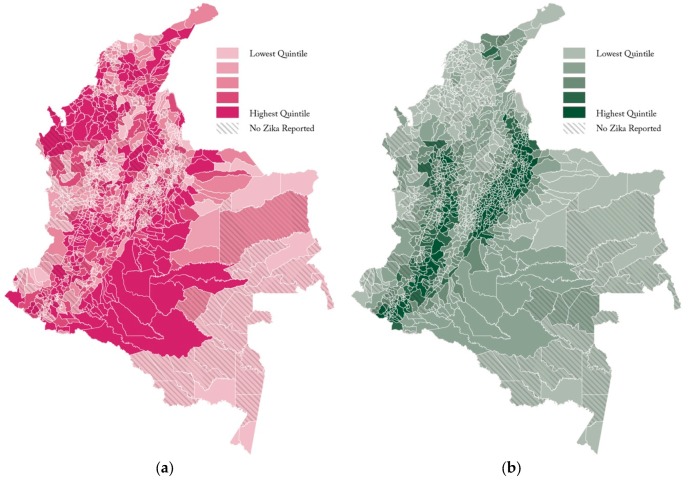
Municipality-level cropland linear density measure (**a**), and average elevation (**b**).

The non-land use variables were also important to our models. Population density had substantial effect on the amount of ZIKV in a region. Each additional 100 persons per square kilometer increased the counts by an amount ranging from 34.5 to 45.4 percent. Changes in per capita GDP were insignificant for two models and showed a slightly negative relationship in the other two. Increases in elevation made substantial differences in ZIKV cases, with an increase in 1000 m decreasing reported ZIKV by 22.4 to 32.9 percent in Columbia. We show the metric of the linear density of cropland and the average elevation in Columbia in Figure 3.

## 4. Discussion

The linear density of Grassland and Cropland land use boundaries exhibit a positive association with ZIKV transmission in Colombia during 2016. Evaluating such findings through the lens of land development plans could aid in the identification of potential hotspots. Identification of these high-risk areas will inform policy changes to reduce the geometric complexity of land use boundaries, thus protect public health by reducing the risk of VBD transmission. While this model was applied specifically to ZIKV data, it could in theory be used to examine other potential emerging zoonotic threats that spillover from sylvatic to urban cycles as land use and boundaries change with deforestation.

Linear density is a product of human behaviors and actions carried out to meet the demands of a growing population. These are often expressed through urban morphology and corresponding rural and urban land development plans and policies [11,17,18,19]. The geometric complexity factor plays a more critical role in determining the magnitude of VBD and land use association than does the relative area (proportion abundance) or patch count (patch density). Rural and urban morphology and land development plans and policies that emphasize simple and compact rather than complex and irregular patch geometry will reduce linear density and thus reduce the spatial breadth over which humans and vectors come in contact [14].

A simple and compact geometry increases a patches core area. This land use boundary related factor, more so than patch area or count, is associated with habitat health and a reduction in vector-to-human interface [39,40]. Larger unbroken patches result in greater control of hosts and vectors by predators, thus naturally controlling VBD outbreaks [41]. While a patch may be large enough to support a given species, it may not contain a core area large enough to support a diverse range of species [42]. Preservation of species diversity reduces the likelihood that pathogens can exploit vacant ecological niches [10,12,13]. 

In addition to core area, changes to rural and urban morphology and land development plans and policies should account for the interplay between land use types. The expansion of Cropland and Grassland, mainly through deforestation in Colombia, for agricultural and pastoral purposes increases their interaction potential. These activities involve the felling of trees, which reduces faunal shade, increases air and water temperatures, and increases the pooling of water. All of these factors promote mosquito breeding and an acceleration of larval development from one to two weeks to as quickly as 4.5 days [13,40]. Similarly, Aedes mosquitoes prefer to inhabit forest edges rather than densely forested areas [20,21]. Therefore, increasing linear density creates more favorable breeding habitats for vectors of ZIKV.

Each 1000-m increase in elevation was associated with an approximately 28 percent decrease in ZIKV risk, a result likely due the influence of lower temperatures on mosquito abundance. While Shragai et al. [41] cite associations between ZIKV risk and wealth in urban areas, our results show a very limited and inconsistent relationship. Our ability to directly investigate urban areas is limited, as Artificial Surface accounts for only 0.011 percent of land use in Colombia as depicted in GLC-SHARE.

This research found positive associations between ZIKV transmission in Colombia during 2016 through three separate land use metrics of Grassland and Cropland land use boundaries. The research approach can be applied to current, granular data and to land development plans. The delta between the current and future state of an areas land use will reveal potential hotspots of increased VBD risk. Armed with this information, policy makers can alter land development plans, emphasizing simple and compact rather than complex and irregular land use geometry.

### Limitations

This study sets a framework for future research that could incorporate additional landscape metrics, areas of interest, and other VBDs. Data availability, resolution, and quality factors restricted this study to ZIKV in Colombia during the 2016 outbreak. Research into the same VBD across two disparate study areas would provide a more robust comparison that improves the assessment of spatial association patterns across space. This research includes the uncertainty inherent with the investigation of a single year snapshot from one geographic region (Colombia—2016). Research that utilizes data spanning multiple years across multiple study areas can improve the assessment of spatial association patterns over time. Such an endeavor could also evaluate differences in ZIKV risk, which might be lower in a more developed country compared to Colombia’s rural expanding settlements due to vector density, infrastructure for waste management, and improved housing structures.

Research outcomes could be further refined through incorporation of additional landscape measurement methods, such as perimeter-area methods to assess fractal dimension, core area methods to assess the core to edge ratio, and contrast methods to assess the magnitude of difference in land use along patch edges [39]. Future research with more granular satellite data may be useful in creating risk maps for use by local public health officials. Such a map could be used to identify areas with parameters that are conducive to transmission that have zero cases reported and suggest that expanded serosurveillance or xenosurveillance be deployed to confirm the absence of cases. Finally, more granular data would enable the determination of whether there was a minimum land use patch size that is linked to higher rates of ZIKV. 

The use of second-order administrative units to evaluate land use and VBD associations offers benefits and drawbacks. Spatial granularity reveals associations hidden at state, regional, or national scales [25]. Conversely, uncertainty arises when VBD transmission occurs outside of the unit where the patient receives health care. Similar to the transmission/report uncertainty, the location of labs and clinics in relation to the patient seeking a diagnosis introduces uncertainty. Factors such as drive time, convenience, and insurance could influence a patient’s decision to seek medical attention outside of the VBD transmission unit [42]. However, VBD occurrence data that includes the probable transmission location would allow for an increase in the spatial granularity of analysis, perhaps at the sub-municipality level. While these uncertainties and limitations, and the exclusion of complex disease ecology and dynamics, hinder the accurate identification disease hotspots at the sub-municipality level, the evaluation of land development plans using the methodology described within this research will reveal municipalities that could experience greater risk of VBD.

## 5. Conclusions

An opportunity exists to focus public health attention on potential VBD hotspots, thus mitigating the impact of VBD through integrated approaches of land development planning, zoning, and policies. Approaches that emphasize the simplification of patch shape geometry will reduce land use linear density within a given area, potentially resulting in an associated decrease in VBD occurrence. Such actions applied to Grassland land use will reduce ZIKV occurrence in Colombia. Through the lens of health and ecology, policy/decision makers within local governments can reduce future VBD occurrences through changes in land development that accounts for VBD risk. Such proactive risk avoidance will reduce the social and economic impacts of potential VBD outbreaks, along with the burden borne by local health agencies. Consideration of VBD risk based on the impact of land development plans represents a shift in urban morphology practices and the corresponding ecological modifications. The public health community can supplement its prevention and mitigation toolbox through the spatial analytic methods described herein to identify areas that could experience an increase in VBDs and possibly other zoonotic diseases linked to land use boundaries and fragmentation.

## Figures and Tables

**Table 1:** Mapping of GLC-SHARE land use classes to Research Classes.

GLC-SHARE Class	Research Class
Artificial Surface	Artificial Surface
Cropland	Cropland
Grassland	Grassland
Shrub-Covered	
Sparse	
Herbaceous	
Tree-Covered	Tree-Covered
Mangrove	Waterlogged
Bare Soil	Bare Soil
Water Bodies	Water Bodies
Snow and Glacier	Snow and Glacier

**Table 2:** Variable information for each landscape metric at the municipality level.

	Variable	Min	Max	Mean	Standard Deviation
	ZIKV Occurrence (Cases)	0.000	72,900.000	447.788	2982.191
	Population Density (People/Sq. KM)	0.234	31,754.989	402.433	1715.979
	Per Capita GDP (Annual Income/Person)	984.425	889,550.410	12,033.233	32,643.741
	Average Elevation (Meters)	2.990	6167.250	1326.178	1006.648
**Linear Density**	Cropland (KM/Sq. KM)	0.000	1.523	0.334	0.311
	Grassland (KM/Sq. KM)	0.000	1.419	0.398	0.292
**Proportion Abundance**	Cropland (Percent)	0.000	93.553	12.735	17.719
	Grassland (Percent)	0.000	100.000	21.356	23.994
**Patch Density**	Cropland (Percent)	0.000	88.889	30.043	19.441
	Grassland (Percent)	0.000	100.000	38.223	20.086

Sq. KM—Square kilometer.

**Table 3:** Negative binomial regression results for ZIKV.

Variable		Model 1		Model 2		Model 3		Model 4	
		IRR	95% CI	IRR	95% CI	IRR	95% CI	IRR	95% CI
Constant		258.428	222.378, 300.323	511.820	432.073, 606.285	208.514	162.733, 267.175	197.773	151.958, 257.401
Population Density (People/Sq. KM) *		1.345	1.297, 1.394	1.462	1.403, 1.524	1.454	1.398, 1.512	1.407	1.353, 1.464
Per Capita GDP (Annual Income/Person) **		0.995	0.992, 0.998	1.002	0.998, 1.007	1.002	0.998, 1.006	0.995	0.993, 0.998
Average Elevation (Meters) **		0.329	0.303, 0.357	0.304	0.280, 0.329	0.224	0.206, 0.243	0.282	0.259, 0.307
**Linear Density**	Cropland	1.001	1.000, 1.002					1.006	1.005, 1.008
	Grassland	1.006	1.005, 1.007					1.002	1.001, 1.003
**Proportion Abundance**	Cropland			0.987	0.983, 0.991			0.974	0.970, 0.978
	Grassland			1.010	1.006, 1.014			0.999	0.996, 1.002
**Patch Density**	Cropland					0.998	0.994, 1.002	0.995	0.991, 0.999
	Grassland					1.035	1.030, 1.039	1.023	1.018, 1.027
**Model Diagnostics**									
*N*		1121		1121		1121		1121	
Pearson Chi-Square		17,753.5		22,429.3		22,437.1		17,230.9	
Likelihood Ratio Chi-Square		2268.1 (0.001)		1749.5 (0.001)		1996.6 (0.001)		2522.2 (0.001)	
AIC		13,674.3		14,192.9		13,945.8		13,428.2	

* Unit is scaled by 100; ** Unit is scaled per 1000.

## Supplementary Materials

The following are available online at https://www.mdpi.com/1660-4601/17/4/1127/s1, Table S1: Correlation Matrix of Model Independent Variables.
