# Supplementary material for: Spatial Associations Between Land Use and Infectious Disease: Zika Virus in Colombia
Source: Int J Environ Res Public Health. 2020 Feb 11;17(4):1127. doi: 10.3390/ijerph17041127 (PMC7068401; doi:10.3390/ijerph17041127)
Supplement: Supplementary file 1 [file ijerph-17-01127-s001.pdf]

International Journal of Environmental Research and Public Health

MDPI

Supplementary Table S1. Correlation Matrix of Model Independent Variables.

		Pop Density	GDP Per Capita	Avg Elevation	Linear Density		Proportion Abundance		Patch Density	
					Cropland	Grassland	Cropland	Grassland	Cropland	Grassland
Pop Density		X								
GDP Per Capita		0.001	X							
Avg Elevation		0.033	-0.052	X						
Linear Density	Cropland	-0.053	0.021	-0.221	X					
	Grassland	-0.037	0.256	-0.187	0.250	X				
Proportion Abundance	Cropland	0.006	-0.006	0.195	0.211	-0.074	X			
	Grassland	0.134	0.113	-0.110	-0.027	0.192	-0.070	X		
Patch Density	Cropland	-0.080	-0.026	0.081	0.162	-0.148	0.025	-0.108	X	
	Grassland	-0.013	-0.036	0.184	-0.068	0.022	-0.098	-0.166	-0.394	X
